# Targeting proteostasis maintenance and autophagy in senescence

**DOI:** 10.18632/aging.203941

**Published:** 2022-03-07

**Authors:** Valentin L’Hôte, Carl Mann, Jean-Yves Thuret

**Affiliations:** 1 Université Paris-Saclay, CEA, CNRS, Institute for Integrative Biology of the Cell (I2BC), Gif-sur-Yvette, France

**Keywords:** cellular senescence, senolytics, proteostasis, selective autophagy, BRAF-V600E

The pharmacological elimination of senescent cells by the means of selectively toxic compounds, termed senolytics, is a strategy that has gained increasing interest in the last ten years as a potential means to alleviate age-related diseases, as well as to potentiate chemotherapy and to prevent malignant transformation. Senescence is a cellular response to stress, alternative to apoptosis, in which the cell undergoes a quasi-irreversible proliferative arrest accompanied by important modifications of its transcriptional programs, and often secretes a collection of inflammatory factors termed the senescence-associated secretory phenotype (SASP). Senescence-inducing stressors are various and notably include telomere attrition, chemotherapeutic agents, ionizing radiations, oxidative stress, and oncogene activation. Senescent cells accumulate during aging and in many pathological contexts, where they play a detrimental role often owing to SASP-mediated chronic inflammation. In many pathological conditions in which accumulating senescent cells are implicated, their elimination by senolytics was shown to be highly beneficial.

The oncogenic V600E mutation in the BRAF kinase induces senescence in melanocytes and leads to the formation of nevi (moles). Melanoma can arise from BRAF-V600E melanocytic nevi, suggesting mechanisms of senescence escape at play. One strategy to prevent melanoma formation in patients at risk would be to eliminate pre-malignant, senescent melanocytes, with senolytics.

In a recent study, we sought to uncover novel vulnerabilities of BRAF-V600E-induced senescent cells (BRafSen cells) that could be targeted by senolytics to prevent melanoma formation [1]. To this end, we employed a fibroblast senescence model of ectopic BRAF-V600E expression. We found that cardioglycosides, such as ouabain, showed exceptional senolytic potency in BRafSen fibroblasts. Interestingly, we found that BRAF-V600E expression induced endoplasmic reticulum stress and a subsequent increase in basal autophagy flux in senescent cells. We demonstrated that ouabain inhibited autophagy through Na,K-ATPase signal transduction, and that BRafSen cells required this heightened autophagy flux for survival, explaining their high sensitivity to cardioglycosides. Accordingly, inhibiting autophagy through other routes, such as with chloroquine or bafilomycin A1, also led to senolysis in BRafSen cells.

The relationship between cellular senescence and autophagy is regarded as paradoxical. Autophagy activation in response to stress can successfully resolve it and thus spare the cell from entering senescence. Aiming to identify ways of specifically eliminating cancer cells, Schepers et al. recently showed that targeting macroautophagy by ULK1 inhibition induces senescence in a panel of cancer cells (but not in normal BJ fibroblasts), and renders cells sensitive to senolysis by BH3-mimetic ABT-263 [2]. However, if a cell does commit to senescence by other ways, autophagy becomes essential for cell survival and senescence establishment. Indeed, SASP production imposes its burden on the secretory pathway, calling for increased proteostasis maintenance. In this regard, the first-ever demonstration of selective pharmacological elimination of senescent cells consisted in depriving therapy-induced senescent lymphoma of adaptive autophagy, leading to proteotoxic stress overload due to SASP expression [3]. Senolysis can actually be achieved by modulating autophagy in either direction: inhibiting autophagy can lead to proteotoxic stress in senescent cells producing an abundant SASP [1] [3], and conversely, further activating autophagy can selectively kill senescent cells through type II autophagic cell death, i.e. excessive “self-eating” [4]. On the other side of the proteostasis network, inhibitors of HSP90 chaperone proteins were found to be senolytic early on. HSP90 activity was essential in senescence to stabilize activated phospho-AKT, which is a master regulator of senescent cell survival [5].

Beyond bulk autophagy flux modulations to cope with increased secretory demands, finer processes appear to be at play in regulating proteostasis in senescence. Recently, it was shown that the stability of a defined set of proteins was regulated by selective autophagy in senescence through differential interactions with ATG8 family receptors. This selective autophagy network was fundamental in shaping several facets of the senescent phenotype, including redox homeostasis (through KEAP1 degradation), SASP production (through TNIP1 degradation), and proteostasis (through eIF3 degradation) [6]. In another study, nuclear selective autophagy was shown to mediate the degradation of SIRT1 in aging and in senescence via LC3, participating in the expression of SASP components otherwise downregulated by SIRT1 [7]. These studies thus pave the way for a more precise understanding of autophagy regulation in the physiology and the proteostasis of senescent cells, and the discovery of potentially more potent senolytic strategies through the targeting of distinct components of the selective autophagy network (Figure 1). The generality of these autophagic pathways in different senescent states also remains to be explored. Variable levels of proteostatic stress in different senescent states, and potential specificity in the use of particular autophagy pathways in different cell types may allow selective elimination of specific types of senescent cells. This may be beneficial in the light of recent work suggesting that indiscriminate removal of senescent cells may be harmful [8].

**Figure 1:**
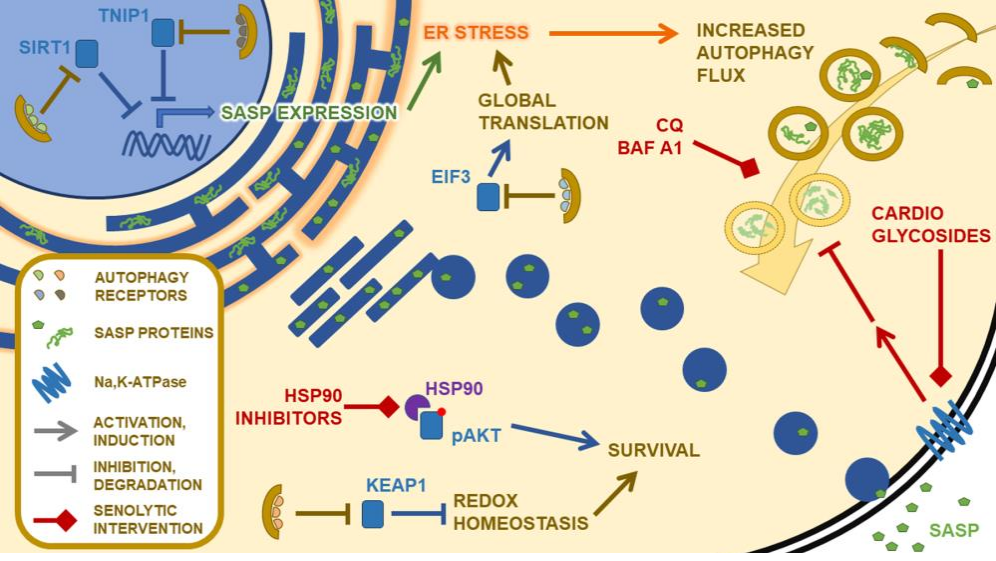
Targeting the proteostasis network in senescence.

## References

[1] L’Hôte V, et al. Aging Cell. 2021; 20:e13447. 10.1111/acel.13447 34355491 PMC8564827

[2] Schepers A, et al. Mol Cancer Res. 2021; 19:1613–21. 10.1158/1541-7786.MCR-21-0146 34158393 PMC7611779

[3] Dörr JR, et al. Nature. 2013; 501:421–25. 10.1038/nature12437 23945590

[4] Wakita M, et al. Nat Commun. 2020; 11:1935. 10.1038/s41467-020-15719-6 32321921 PMC7176673

[5] Fuhrmann-Stroissnigg H, et al. Nat Commun. 2017; 8:422. 10.1038/s41467-017-00314-z 28871086 PMC5583353

[6] Lee Y, et al. Dev Cell. 2021; 56:1512–1525.e7. 10.1016/j.devcel.2021.04.008 33915088

[7] Xu C, et al. Nat Cell Biol. 2020; 22:1170–79. 10.1038/s41556-020-00579-5 32989246 PMC7805578

[8] Grosse L, et al. Cell Metab. 2020; 32:87–99.e6. 10.1016/j.cmet.2020.05.002 32485135

